# Impact of adjuvant chemotherapy on survival in ypT0-2 N0 rectal cancer

**DOI:** 10.1007/s00384-024-04781-x

**Published:** 2025-01-03

**Authors:** Mohamed Osama Alorabi, Abdelrahman Gouda, Mohammed Abdeen, Ahmed Said, Moamen Abdelaal, Reem Eid, Maha Yahia

**Affiliations:** 1 Clinical Oncology Department, Faculty of Medicine, Ain Shams University, Cairo, 11591 Egypt; 2 Clinical Oncology Department, Faculty of Medicine, Aswan University, Aswan, Egypt; 3 Clinical Oncology Department, Faculty of Medicine, Cairo University, Giza, Egypt; 4 Radiation Oncology Department, National Cancer Institute, Cairo University, Giza, Egypt; 5 Biostatics & Epidemiology Department, National Cancer Institute, Cairo University, Giza, Egypt; 6 Medical Oncology Department, National Cancer Institute, Cairo University, Giza, Egypt; 7 Medical Oncology Department, Shefa Al Orman Cancer Hospital, New Tiba City, Egypt; 8 Radiation Oncology Department, Shefa Al Orman Cancer Hospital, New Tiba City, Egypt; 9 Clinical Nutrition Department, Shefa Al Orman Cancer Hospital, New Tiba City, Egypt

**Keywords:** Rectal adenocarcinoma, Downstaginging, Pathological response, Survival outcomes

## Abstract

**Purpose:**

The role of adjuvant chemotherapy in rectal cancer patients downstaged to ypT0-2 N0 after neoadjuvant chemoradiotherapy (CRT), and surgery is still debated. This study investigates the impact of adjuvant chemotherapy on survival outcomes in this patient population.

**Methods:**

This retrospective study analyzed hospital records of rectal cancer cases from Shefa Al Orman Cancer Hospital between January 2016 and December 2020, focusing on patients downstaged to ypT0-2 N0 after neoadjuvant CRT and surgery. Patients were divided into two groups based on whether they received adjuvant chemotherapy. Baseline characteristics, DFS, and OS were compared, and survival factors were analyzed using univariate and multivariate Cox regression.

**Results:**

Eighty-five patients met the inclusion criteria; 55 received adjuvant chemotherapy, and 30 did not. The median age was 52, but those receiving adjuvant therapy were younger (47 vs. 60 years, *P* = 0.006). No significant differences were observed in sex, tumor location, or pathology between groups. Although adjuvant chemotherapy showed a trend toward better 3-year DFS (89.5% vs. 81.9%, *P* = 0.153) and OS (88.1% vs. 84.6%, *P* = 0.654), these differences were not statistically significant. Univariate and multivariate analyses confirmed no significant effect of adjuvant chemotherapy on DFS or OS, nor were any other variables significantly associated with survival.

**Conclusion:**

Adjuvant chemotherapy did not significantly improve DFS or OS in rectal cancer patients downstaged to ypT0-2 N0 following neoadjuvant CRT and surgery. Further studies are needed to define the role of adjuvant therapy in this group.

**Supplementary Information:**

The online version contains supplementary material available at 10.1007/s00384-024-04781-x.

## Introduction

Among cancers, colorectal cancer (CRC) ranks 3rd in males and 2nd d in females, making it a serious issue in world health. Males are more likely to develop and die from colorectal cancer than females, and the disease makes up around 10% of all cancer cases globally, according to the GLOBOCAN database [1]. In Egypt, CRC accounts for around 3.9% of all cancer cases, ranking seventh in the area [2].

For patients with locally advanced rectal cancer (LARC), surgical intervention stands as the cornerstone of treatment. However, rectal cancer is more prone to local recurrence than colon cancer, highlighting the importance of neoadjuvant chemoradiotherapy in curative treatment plans [3, 4]. The conventional treatment for LARC (T3-4N-ve or T1-4N + ve) involves neoadjuvant CRT and total mesorectal excision (TME) and, in many cases, adjuvant chemotherapy with fluoropyrimidine with or without oxaliplatin. Neoadjuvant treatment has been shown to improve sphincter preservation and promote tumor downstaging [5, 6]. Furthermore, ypT0N0, or a pathologic complete response (pCR), is attained by about 15% of patients and is linked to excellent long-term survival results [7, 8]. Twenty percent of patients also get tumor downstaging to ypT1/T2N0 after neoadjuvant treatment [9].

Despite these advances, about 1/3 of rectal cancer patients eventually develop distant metastasis after surgery [10]. One possible solution to this problem is adjuvant chemotherapy, which can eradicate micrometastases, decrease recurrence, and improve survival [11–13]. The benefit of adjuvant chemotherapy in ypT0-2N0 disease following neoadjuvant CRT and surgery is still debatable, as it does not always achieve positive results [14–17]. The National Comprehensive Cancer Network (NCCN) states that adjuvant chemotherapy decisions should depend on pre-treatment staging rather than post-surgical pathology. This uncertainty, combined with low compliance with the NCCN guidelines, reflects the complexity of decision-making in this setting [18, 19].

This research aims to evaluate the effect of adjuvant chemotherapy on the survival of rectal cancer cases who were downstaged to ypT0-2N0 after neoadjuvant CRT and surgical intervention.We believe our study provides significant value for several reasons. First, while the practice of total neoadjuvant therapy (TNT) is gaining traction globally, its implementation remains highly variable across institutions, as evidenced by recent surveys from Germany and China showing diverse treatment protocols and practices [20, 21]. This variability underscores the importance of continuing research to refine therapeutic strategies for rectal cancer. Importantly, growing evidence suggests that some rectal cancer patients achieving ypT0-2N0 after neoadjuvant chemoradiation may fare well without additional chemotherapy, potentially avoiding overtreatment and the associated treatment-related toxicities [9, 22]. This is particularly relevant for elderly and frail patients with limited tolerance for intensive treatment regimens. Rather than adopting a "one-size-fits-all" approach with TNT, further research is crucial to identify patient subgroups who could benefit from de-escalated treatment strategies. Moreover, our study is the first to address this topic in the Egyptian, Arab, or African context, where healthcare infrastructure and treatment paradigms differ significantly from those in high-income countries. As no regional data currently exists, our findings can serve as a benchmark for future research and guide clinical practice in similar settings. We hope this perspective highlights the relevance of our study in enriching the global discourse on rectal cancer management and personalized treatment approaches.

## Study population and methodology

### Study design

This retrospective analysis was conducted at Shefaa El Orman Cancer Hospital and concentrated on patients with rectal adenocarcinoma from January 2016 to December 2020. The Institutional Review Board (IRB) at Shefaa El Orman Cancer Hospital approved the study, which was conducted according to the Declaration of Helsinki. Because of the retrospective nature of the study, informed consent was waived. However, patient confidentiality was protected by anonymizing all data that were securely stored and accessed only by authorized personnel. All analyses were performed on aggregated, anonymized data to maintain confidentiality.This study adhered to the Strengthening the Reporting of Observational Studies in Epidemiology (STROBE) guidelines for reporting observational studies. The completed STROBE checklist is included as supplementary material.

### Criteria for participant selection

Patients had to be at least 18 years old and diagnosed with rectal adenocarcinoma confirmed histologically to be eligible to participate. They had to have completed neoadjuvant concurrent chemoradiotherapy (CRT) and radical surgical resection, and they had to have an ECOG performance level of 0–2. Inclusion in the study was restricted to patients who had a pathological staging of ypT0-2N0 based on postoperative pathological examination of the surgical specimen.

The sample size was determined by the number of eligible patients available during the study period, ensuring adequate power for detecting clinically significant differences.

### Data retrieval process

Data were retrieved retrospectively from hospital medical records, including both demographic and clinical characteristics. To minimize bias, consistent data collection procedures were followed, and only validated clinical records were used. Patient anonymity was maintained throughout the study by removing all identifiable information. Selection bias was minimized by including all eligible patients meeting the inclusion criteria during the study period. Demographic data included age, sex, smoking status, and family history of cancer. Clinical data collected comprised ECOG performance status, comorbidities (such as hypertension and diabetes), tumor-related details (e.g., tumor grade, distance from the anal verge), initial carcinoembryonic antigen (CEA) levels, and pretreatment clinical staging (both T and N stages).

Treatment-related data were also collected, including details on neoadjuvant CRT, the type of surgery performed (abdominoperineal resection [APR] or low anterior resection [LAR]), and the surgical technique used (laparoscopic or open surgery). The administration of adjuvant chemotherapy was also recorded, including the type of regimen used (capecitabine or CAPOX/FOLFOX). Follow-up data were obtained on local recurrence, distant metastasis, and survival.

Data on certain risk factors, such as extramural venous invasion, tumor deposits, and circumferential margin status, were not consistently available due to limitations in preoperative imaging and postoperative histopathology reporting practices at the time of this study. Missing data for key variables were assessed. If data were missing, they were handled using listwise deletion to ensure accuracy in analysis.

### Treatment protocols

Patients received neoadjuvant concurrent chemoradiotherapy (CRT) with either three-dimensional conformal (3DCRT) or intensity-modulated (IMRT) radiotherapy in combination with oral capecitabine. The prescribed radiation dose was 45–50.4 Gy in 25–28 daily fractions over five weeks. Patients concurrently received capecitabine 825 mg/m^2^ twice daily during radiotherapy days. Surgical resection was performed 4 to 12 weeks after the end of CRT. The type of surgery—either low anterior resection (LAR) or abdominoperineal resection (APR)—was chosen depending on tumour location and patient factors. All patients had total mesorectal excision (TME) as part of the surgical procedure. Based on clinical judgment, adjuvant chemotherapy was recommended in selected patients following surgery. Adjuvant chemotherapy, usually given for four months after surgery, protocols included either capecitabine (1250 mg/m^2^ twice daily on days 1–14, repeated every 21 days) or CAPOX/FOLFOX. The CAPOX protocol included intravenous oxaliplatin (130 mg/m^2^ on day 1) and oral capecitabine (1000 mg/m^2^ every 12 h on days 1–14, repeated every 21 days). The FOLFOX regimen consisted of intravenous oxaliplatin (85 mg/m^2^), leucovorin (400 mg/m^2^), and 5-fluorouracil (5-FU), followed by a continuous 46-h infusion of 2400 mg/m^2^ 5-FU, repeated every two weeks.

In line with hospital policy, every treatment decision was thoroughly reviewed and discussed during a multidisciplinary team (MDT) meeting. These meetings involved input from various medical professionals, including oncologists, surgeons, radiologists, pathologists, and other relevant specialists, ensuring a comprehensive and collaborative approach to patient care. The decision to administer adjuvant chemotherapy and the choice of regimen (capecitabine vs. CAPOX/FOLFOX) were determined during multidisciplinary team (MDT) meetings. These decisions were based on patient-related factors such as age, comorbidities, and performance status, as well as tumor characteristics and clinical response to neoadjuvant therapy.

### Monitoring and evaluation of patient outcomes

After completing therapy, patients had to attend regular follow-up appointments. These visits were planned every 3 months for the first 2 years, every 6 months up to 5 years, and then annually. Physical exams, carcinoembryonic antigen (CEA) tests, and imaging procedures like chest, liver, and pelvis CT scans were all part of each patient's routine assessments. A colonoscopy was performed every 3–5 years after one year, provided no abnormalities were detected.

### Statistical analysis

All statistical analyses were performed using IBM SPSS Statistics, version 26. Continuous variables were summarized as means and standard deviations (SD) for normally distributed data, or as medians and interquartile ranges (IQR) for non-normally distributed data. Categorical variables were presented as frequencies and percentages. Comparisons between groups were made using chi-square or Fisher’s exact tests for categorical variables, and continuous variables were analyzed using Mann–Whitney U tests or independent t-tests as appropriate.

Survival analyses for DFS and OS were conducted using Kaplan–Meier methods, with differences between groups compared using the log-rank test. DFS was defined as the time from surgery to either local or distant recurrence, while OS was defined as the time from diagnosis to death from any cause or last follow-up. Multivariate analysis was performed using the Cox proportional hazards regression model to identify independent prognostic factors associated with DFS and OS. Hazard ratios (HR) and 95% confidence intervals (CI) were reported. A p-value of ≤ 0.05 was considered statistically significant for all analyses.

The findings were compared with prior studies to assess consistency and enhance validity. Differences were critically analyzed in the discussion section.

## Results

### Clinical and tumor characteristics

The study reviewed the clinicopathological features of 85 rectal cancer patients treated with neoadjuvant chemoradiotherapy (CRT) and surgery, as presented in Table 1, 55 of whom received adjuvant chemotherapy, while 30 did not. Key findings revealed that age was a significant factor in the decision to receive adjuvant chemotherapy, with the median age of patients in the no-adjuvant group being significantly higher (60 years, IQR 48–64.75) compared to those who received adjuvant therapy (47 years, IQR 40–57.5; *P* = 0.006). Gender distribution, tumor location, grade, initial CEA levels, clinical T and N stages, and type of surgery were balanced between the groups, suggesting no systematic differences in these characteristics. Tumors were predominantly located in the middle rectum (54.1%), and most were grade II (90.6%). Clinical staging showed that 65.88% were T3, and 54.12% were N1. Surgical approaches included low anterior resection (LAR) in 60% of cases and abdominoperineal resection (APR) in 40%, with no significant difference between groups. Open surgery was more common (74.12%) than laparoscopic surgery (25.88%), and this distribution was consistent across the groups. Among the 85 patients included in the study, 33 (37.5%) achieved ypT0N0, 4 (4.5%) ypT1N0, and 48 (58%) ypT2N0. These proportions highlight the significant representation of ypT0N0 cases in our cohort.

**Table 1:** Patient profile and tumor characteristics

Characteristic	Total (*n* = 85)	Adjuvant Chemotherapy (*n* = 55)	No Adjuvant Chemotherapy (*n* = 30)	*P*-value
Age in years (median, IQR)	52 (42–61)	47 (40–57.5)	60 (48–64.75)	0.006*
Male (%)	39 (45.9%)	26 (47.3%)	13 (43.3%)	0.728
Female	46 (54.12%)	29 (52.73%)	17 (56.67%)	0.728
Lower rectum (0–5 cm)	34 (40%)	22 (40%)	12 (40%)	0.753
Middle rectum (6–10 cm)	46 (54.1%)	29 (52.7%)	17 (56.7%)	0.753
Upper rectum (11–15 cm)	5 (5.9%)	4 (7.3%)	1 (3.3%)	0.753
Grade I	1 (1.2%)	0 (0%)	1 (3.3%)	0.352
Grade II	77 (90.6%)	51 (92.7%)	26 (86.7%)	0.352
Grade III	7 (8.2%)	4 (7.3%)	3 (10.0%)	0.352
Initial CEA < 5 ng/mL	22 (25.9%)	13 (23.6%)	9 (30%)	0.796
Initial CEA ≥ 5 ng/mL	13 (15.3%)	9 (16.4%)	4 (13.3%)	0.796
Unknown CEA	50 (58.8%)	33 (60%)	17 (56.7%)	0.796
Clinical T2	11 (12.94%)	5 (9.09%)	6 (20%)	0.462
Clinical T3	56 (65.88%)	38 (69.09%)	18 (60%)	0.462
Clinical T4	17 (20%)	11 (20%)	6 (20%)	0.462
Unknown Clinical T status	1 (1.18%)	1 (1.82%)	0 (0%)	0.462
Clinical N0	9 (10.59%)	6 (10.91%)	3 (10%)	0.759
Clinical N1	46 (54.12%)	31 (56.36%)	15 (50%)	0.759
Clinical N2	29 (34.12%)	17 (30.91%)	12 (40%)	0.759
Unknown Clinical T status	1 (1.18%)	1 (1.82%)	0 (0%)	0.759
LAR	51 (60%)	30 (54.55%)	21 (70%)	0.165
APR	34 (40%)	25 (45.45%)	9 (30%)	0.165
Open Surgery	63 (74.12%)	13 (23.64%)	9 (30%)	0.522
Laparoscopic	22 (25.88%)	42 (76.36%)	21 (70%)	0.522
Median Nodal Harvest (median, IQR)	13 (10–16)	14 (11–17)	12 (9–15)	0.118
CRM Involvement (%)	8% (7 patients)	7.3% (4 patients)	10% (3 patients)	0.482
Median Follow-Up (months)	46 months (IQR: 36–58)	45 months (IQR: 36–55)	44 months (IQR: 32–50)	0.645

### Treatment characteristics

Among the 55 patients receiving adjuvant chemotherapy, 29 were treated with Capecitabine and 26 with CAPOX/FOLFOX. The median time from surgery to initiation of adjuvant chemotherapy was comparable between the groups: 5 weeks (IQR: 3–7) for Capecitabine and 4 weeks (IQR: 2.5–6.5) for CAPOX/FOLFOX. Most patients completed the recommended 5–6 cycles of chemotherapy, with 75.9% in the Capecitabine group and 80.7% in the CAPOX/FOLFOX group. A small percentage received fewer cycles, with 20.7% in the Capecitabine group completing 3–4 cycles compared to 7.7% in the CAPOX/FOLFOX group. Only one patient (3.4%) in the Capecitabine group completed 1–2 cycles, while none in the CAPOX/FOLFOX group had fewer than 3 cycles. These findings as illustrated in Table 2 indicate that the majority of patients adhered well to the chemotherapy regimens, with minimal delays in initiating treatment after surgery.

**Table 2:** Adjuvant chemotherapy treatment characteristics

Characteristic	Adjuvant Chemotherapy (*n* = 55)	Capecitabine (*n* = 29)	CAPOX/FOLFOX (*n* = 26)
The time between surgery and the start of chemotherapy in weeks (median, IQR)	4.71 (3–7)	5 (3–7)	4 (2.5–6.5)
1–2 cycles (%)	1 (1.8%)	1 (3.4%)	0 (0%)
3–4 cycles (%)	8 (14.5%)	6 (20.7%)	2 (7.7%)
5–6 cycles (%)	43 (78.2%)	22 (75.9%)	21 (80.7%)

### Treatment outcomes

#### Local recurrence/distant metastasis

The incidence of local recurrence and distant metastasis based on adjuvant chemotherapy shows distinct trends as shown in Table 3. Local recurrence occurred in 3 patients (3.5%) overall, with a lower rate in the adjuvant chemotherapy group (1.8%) versus the no-adjuvant group (6.7%). Distant metastasis was observed in 9 patients (10.6%), with similar rates between the adjuvant chemotherapy group (10.9%) and the no-adjuvant group (10.0%). Liver metastasis occurred in 5 patients (5.9%) overall, with comparable rates between the adjuvant group (5.4%) and the no-adjuvant group (6.7%). Similarly, lung metastasis affected 3 patients (3.5%), with nearly identical rates in both the adjuvant (3.6%) and no-adjuvant groups (3.3%).

**Table 3:** Recurrence and metastasis rates by adjuvant chemotherapy status

Characteristic	Total (*n* = 85)	Adjuvant Chemotherapy (*n* = 55)	No Adjuvant Chemotherapy (*n* = 30)
Local recurrence (%)	3 (3.5%)	1 (1.8%)	2 (6.7%)
Distant metastasis (%)	9 (10.6%)	6 (10.9%)	3 (10.0%)
Liver metastasis (%)	5 (5.9%)	3 (5.4%)	2 (6.7%)
Lung metastasis (%)	3 (3.5%)	2 (3.6%)	1 (3.3%)

**Fig. 1:**
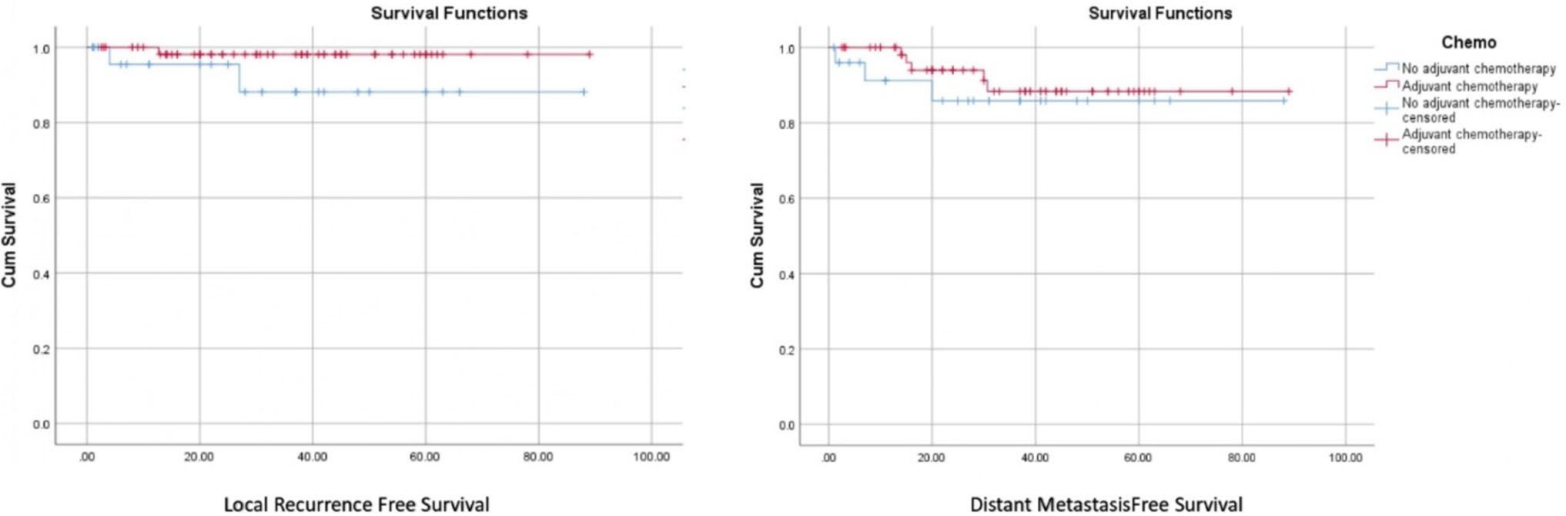
Kaplan–Meier analysis of LRFS and DMFS in ypT0-2 N0 rectal cancer: adjuvant chemotherapy vs. no chemotherapy

Kaplan–Meier analysis showed mean local recurrence-free survival (LRFS) times of 87.56 ± 1.43 months in the adjuvant chemotherapy group and 79.70 ± 5.56 months in the no-adjuvant group (*P* = 0.115). Similarly, mean distant metastasis-free survival (DMFS) times were 81.24 ± 3.28 months and 77.00 ± 5.92 months, respectively (*P* = 0.479). Although adjuvant chemotherapy showed a trend toward improved survival, these differences were not statistically significant as shown in Fig. 1

#### Mortality

The analysis of mortality based on adjuvant chemotherapy status shows notable differences. Overall mortality was observed in 11 patients (12.9%) across the cohort, with a significantly lower rate in the adjuvant chemotherapy group (7.3%) compared to the no-adjuvant chemotherapy group (23.3%). Cancer-related mortality occurred in 7 patients (8.2%), with 2 patients (3.6%) in the adjuvant group and 5 patients (16.7%) in the no-adjuvant group. Non-cancer-related mortality was comparable between the two groups, affecting 4 patients (4.7%) in total, with 2 patients (3.6%) from the adjuvant group and 2 patients (6.7%) from the no-adjuvant group. These findings, as shown in Table 4, suggest that adjuvant chemotherapy may significantly reduce overall and cancer-related mortality in rectal cancer patients.

**Table 4:** Mortality based on adjuvant chemotherapy status

Characteristic	Total (*n* = 85)	Adjuvant Chemotherapy (*n* = 55)	No Adjuvant Chemotherapy (*n* = 30)
Overall mortality (%)	11 (12.9%)	4 (7.3%)	7 (23.3%)
Cancer-related mortality (%)	7 (8.2%)	2 (3.6%)	5 (16.7%)
Non-cancer-related mortality (%)	4 (4.7%)	2 (3.6%)	2 (6.7%)

#### Survival analysis

**Fig. 2:**
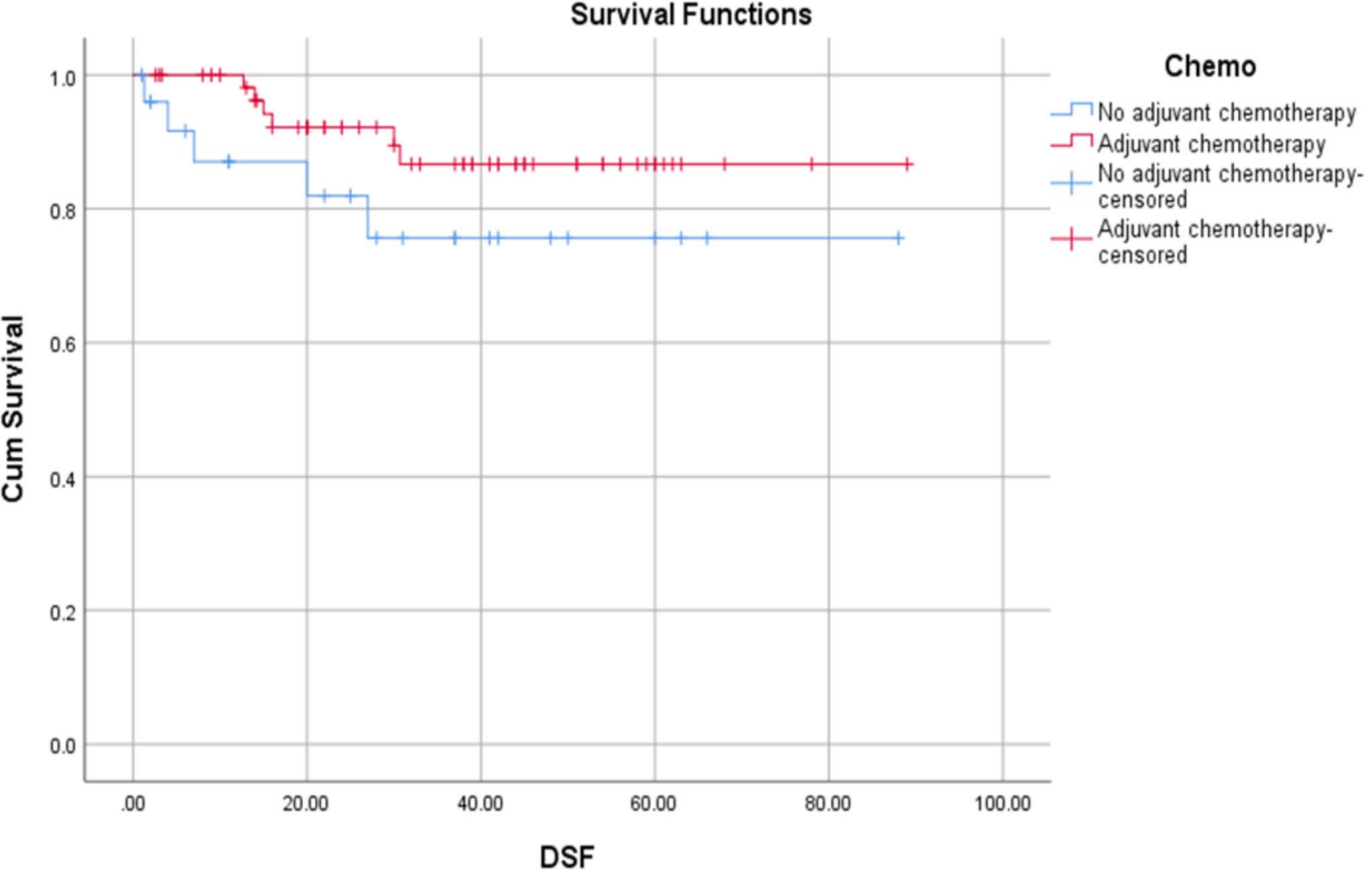
Kaplan–Meier analysis of DFS in ypT0-2 N0 rectal cancer: adjuvant chemotherapy vs. no chemotherapy

The survival analysis compared DFS and OS between the two cohorts. The mean DFS was 69.8 ± 7.16 months in the no-adjuvant chemotherapy group and 79.9 ± 3.46 months in the adjuvant chemotherapy group as shown in Fig. 2. Notably, the median DFS was not reached in either group because more than 50% of patients remained disease-free after the follow-up, indicating favorable outcomes. At the 3-year mark, 81.9% of patients in the no-adjuvant chemotherapy group were disease-free, compared to 89.5% of patients in the adjuvant chemotherapy group. While these numbers suggest a trend toward improved short-term DFS in the adjuvant chemotherapy group, this did not reach statistical significance (*P* = 0.153).

**Fig. 3:**
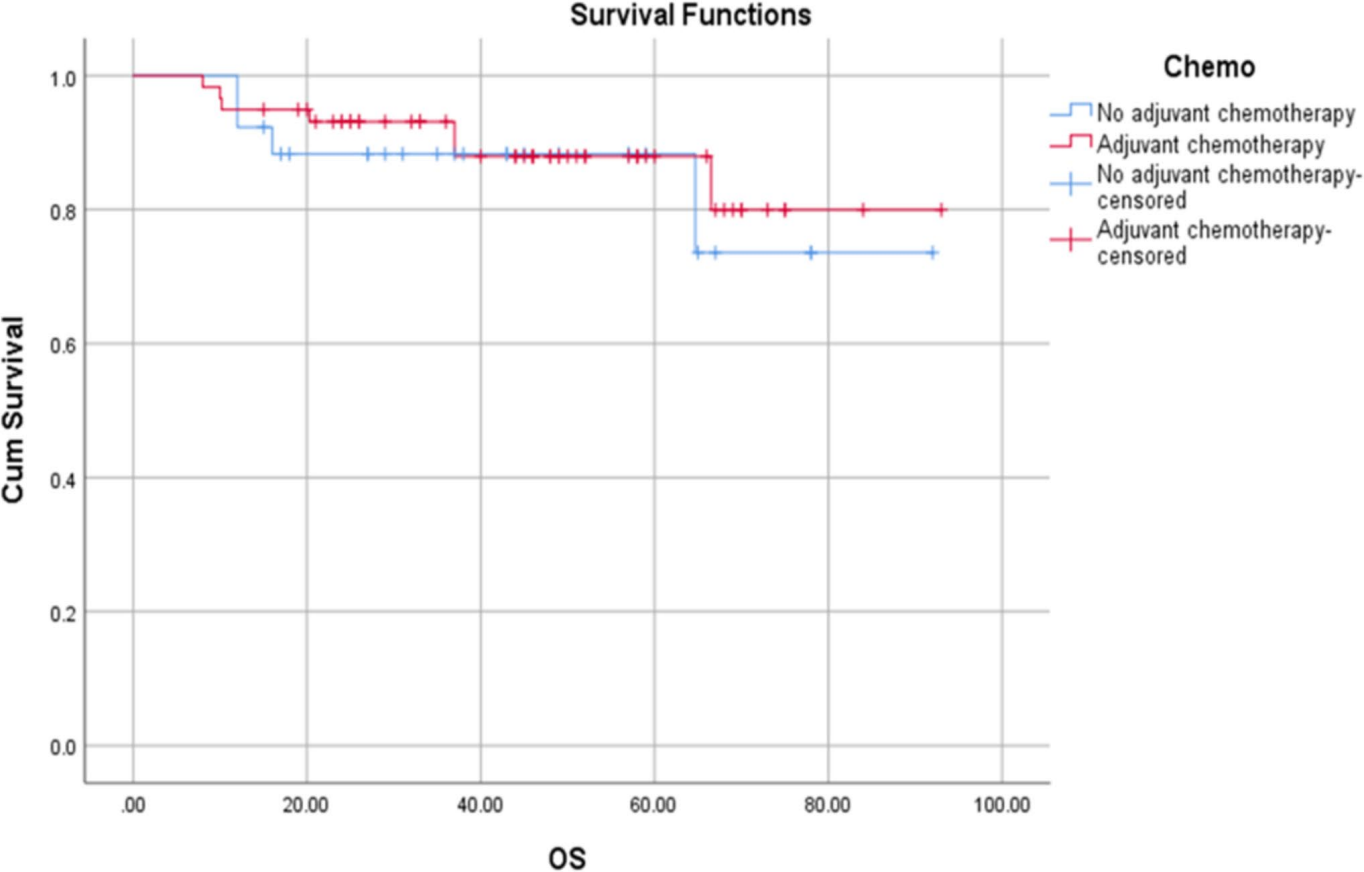
Kaplan–Meier analysis of OS in ypT0-2 N0 rectal cancer with and without adjuvant chemotherapy

Similar trends were observed for overall survival (OS). The mean OS was 78.8 ± 5.96 months with adjuvant chemotherapy and 82.4 ± 3.73 months in the group without adjuvant treatment. Like DFS, the median OS was not reached in either group, reflecting that more than half of the population were still alive after the follow-up period. At 3 years, OS was 84.6% in the no-adjuvant chemotherapy group compared to 88.1% in the adjuvant chemotherapy group. Although there was a slight improvement in survival rates with the use of adjuvant chemotherapy, the difference between the two groups was not statistically significant, as demonstrated by the log-rank test (*P* = 0.654) (Fig. 3).

#### Univariate and multivariate analysis

As presented in Table 5, the univariate and multivariate analyses conducted in this study revealed that none of the assessed clinicopathological factors, including age, gender, tumor location, grade, initial CEA levels, clinical T and N stages, type of surgery, surgical approach, or the use of adjuvant chemotherapy, demonstrated a statistically significant association with disease-free survival (DFS) or overall survival (OS). Notably, adjuvant chemotherapy, hypothesized to improve outcomes, showed no significant effect on DFS (*P* = 0.166 in univariate and *P* = 0.674 in multivariate analyses) or OS (*P* = 0.656 in univariate and *P* = 0.843 in multivariate analyses). Similarly, other factors, such as the higher proportion of T3 tumors and the greater number of patients undergoing abdominoperineal resection (APR) in the adjuvant chemotherapy group, did not significantly influence DFS or OS. These findings suggest that in this cohort, the examined variables, including the receipt of adjuvant chemotherapy, were not significant prognostic factors for survival outcomes.

**Table 5:** Factors influencing OS and DFS: a univariate and multivariate approach

	DFS		OS	
	Univariate Analysis	Multivariate analysis	Univariate Analysis	Multivariate analysis
	*P*	*P*	*P*	*P*
Age (> 50 vs ≤ 50 years)	0.726	0.335	0.466	0.609
Male vs Female	0.331	0.891	0.178	0.876
Lower third vs middle-upper two-thirds tumors	0.116	0.619	0.917	0.943
Grade (II vs III)	0.909	0. 709	0.851	0.749
Initial CEA (≤ 5 vs > 5)	0.968	0.286	0.825	0.520
Initial T status (T2 vs T3 and T4)	0.323	0.847	0.998	0.973
Initial N stage (N0 vs N1 and N2)	0.545	0.483	0.364	1.000
Abdominoperineal vs Lower Anterior Resection	0.969	0.684	0.528	0.546
Open vs. Laparoscopic Surgery	0.372	0.526	0.514	0.643
Adjuvant chemotherapy (Yes vs. No)	0.166	0.674	0.656	0.843

## Discussion

The purpose of this study was to evaluate the effect of adjuvant chemotherapy on disease-free survival (DFS) and overall survival (OS) in patients with downstaged rectal cancer (ypT0-2 N0) following neoadjuvant CRT and radical resection at Shefa Al Orman Hospital. In this group of patients, we found no evidence that adjuvant chemotherapy improved overall survival, disease-free survival, or recurrence rates. Although patients who underwent adjuvant chemotherapy tended toward better survival outcomes, these changes lacked statistical significance. Furthermore, no significant difference was seen between patients who got adjuvant chemotherapy and those who did not in terms of local recurrence-free survival or distant metastasis-free survival. These results support an increasing amount of evidence that downstaged rectal cancer patients with ypT0-2 N0 status may be at risk of overtreatment, and they cast doubt on the use of adjuvant chemotherapy in these patients.

Patients with ypT0N0 are known to have better outcomes than those with residual disease. In our study, 37.5% of patients achieved ypT0N0, representing a substantial proportion of the cohort. Excluding this subgroup would significantly limit the sample size and the generalizability of our findings. Furthermore, including ypT0N0 patients allows us to explore the heterogeneity of response to neoadjuvant chemoradiotherapy and the potential role of adjuvant chemotherapy in different subgroups, contributing to the ongoing debate on optimizing treatment strategies for rectal cancer.

Previous research has also shown no survival benefit from adjuvant chemotherapy in rectal cancer patients who had good histological responses after neoadjuvant treatment, which is in line with our findings. For instance, the European Organization for Research and Treatment of Cancer (EORTC) trial 22921, a landmark randomized phase III trial, assessed the effects of postoperative chemotherapy on patients with rectal cancer who had received preoperative radiotherapy, either with or without chemotherapy. Although the trial did not show a substantial improvement in 10-year OS or DFS, particularly in patients with favorable pathological downstaging, the addition of chemotherapy did improve local control [10, 23]. In our study, patients who achieved ypT0-2 N0 status did not demonstrate a significant survival advantage from adjuvant chemotherapy, indicating that this additional treatment may not be required for patients with minimal residual disease after neoadjuvant CRT.

Similar results have been observed in other trials as well. According to Sainato et al. (2014), in the randomized Italian study, which compared postoperative adjuvant chemotherapy with observation in patients treated with preoperative chemoradiotherapy, there was no significant difference in recurrence rates or overall survival between the two groups. Similarly, the Dutch colorectal PROCTOR/SCRIPT trials, which assessed postoperative fluorouracil/leucovorin or capecitabine in patients with stage II or III rectal cancer, failed to reach full accrual and did not demonstrate a statistically significant difference in survival outcomes between patients and non-recipients for adjuvant chemotherapy [24]. Another study that found no significant increase in survival outcomes with adjuvant chemotherapy was the phase III Chronicle trial in the United Kingdom. This trial compared capecitabine/oxaliplatin with no adjuvant treatment in rectal cancer patients [25]. These results provide more evidence that adjuvant chemotherapy might not help individuals with rectal cancer who had downstaging and ypT0-2 N0 illness.

A meta-analysis of individual patient data from four main trials also determined that fluorouracil-based adjuvant chemotherapy did not enhance OS, DFS, or reduce distant recurrence rates in patients with rectal cancer [26]. Notably, this analysis determined that patients with tumors situated 10 to 15 cm above the anal verge experienced a substantially improved DFS with adjuvant chemotherapy; however, this advantage did not extend to the general rectal cancer population. While we did not find a statistically significant difference in survival rates according to tumor location in our study, this could mean that adjuvant chemotherapy is only helpful for specific subsets of rectal cancer patients, like those with higher tumor locations or worse pathological stages.

Our research is also consistent with the results of the ADORE trial, which assessed the efficacy of oxaliplatin-containing adjuvant chemotherapy in patients with rectal cancer who had undergone neoadjuvant chemoradiotherapy and surgery [27]. In the ADORE trial, patients with ypT3-4 N0 or ypN + disease, who are more advanced than the patients in our research, had a substantially higher 6-year DFS rate in the FOLFOX group. However, our patient type was not included in this benefit. The absence of a survival benefit in our cohort, which consisted solely of patients with ypT0-2 N0 status, implies that adjuvant chemotherapy may not be required in patients who have attained a favorable pathological downstaging following neoadjuvant therapy.

Several studies have demonstrated the prognostic value of pathological response to neoadjuvant CRT in rectal cancer. Park et al. [6] showed that regardless of response category (full, partial, or poor), tumor response to neoadjuvant therapy is a vital early predictor of long-term outcomes, with 5-year recurrence-free survival rates varying significantly. Patients who have complete or almost complete responses, such as those with ypT0-2 N0 disease, typically have excellent long-term results, raising doubts about the necessity of extra adjuvant chemotherapy in these individuals. Our findings reinforce this concept, as patients with ypT0-2 N0 status in our study exhibited favorable survival outcomes without a substantial survival benefit from adjuvant chemotherapy.

Additionally, the value of adjuvant chemotherapy in patients with downstaged rectal cancer has been questioned by several retrospective studies and meta-analyses. In Taiwan, 720 patients with rectal cancer underwent a comprehensive retrospective countrywide investigation by Kuo et al. [28], and they discovered no statistically significant difference in 5-year OS or DFS between patients who received adjuvant chemotherapy and those who did not. Furthermore, no protective benefit of adjuvant chemotherapy was found in stratified analyses, even in patients with more advanced clinical T or N classifications. Liao and his colleagues [29] reported comparable results, following 110 patients with ypT0-2 N0 rectal cancer for a median of 60 months and finding no statistically significant difference in the survival outcomes between patients receiving adjuvant chemotherapy and those not.

Nevertheless, certain studies have demonstrated the potential advantages of adjuvant chemotherapy, particularly in cases of more advanced rectal cancer. Another study offered an intriguing viewpoint, demonstrating that adjuvant chemotherapy was considerably beneficial for patients with ypT2 ypN0, as seen by better OS and DFS compared to no adjuvant treatment. However, Adjuvant chemotherapy did not offer significant survival advantages for patients with ypT0-1 N0 disease. These results indicate that patients with ypT0-1 N0 status may not require adjuvant chemotherapy; however, those with ypT2 N0 may still derive substantial advantages from additional treatment [30].

Seventeen nonrandomized studies involving 4,747 patients with ypT0-2N0 rectal cancer were reviewed in the meta-analysis [31]. According to their findings, patients with ypT0N0 or ypT1-2N0 status did not experience a significant difference in OS, DFS, local recurrence, or distant recurrence following adjuvant chemotherapy. According to the odds ratios for OS (1.53, 95% CI: 0.86–2.72) and DFS (1.22, 95% CI: 0.61–2.42), adjuvant chemotherapy did not significantly improve outcomes. The absence of statistical significance in the recurrence and survival outcomes raises the possibility that routine adjuvant chemotherapy usage in this patient group is not warranted. This meta-analysis supports the idea that patients with rectal cancer who show good pathology responses after neoadjuvant therapy would benefit more from a targeted strategy to adjuvant therapy as opposed to a standard one [31].

Our study has important clinical implications. The lack of a clear survival benefit from adjuvant chemotherapy in patients with ypT0-2 N0 rectal cancer suggests that routine use of adjuvant chemotherapy in this population may be unnecessary. By avoiding overtreatment, we can reduce the toxicity and side effects associated with chemotherapy, which can have a significant impact on patients' quality of life. Furthermore, avoiding unnecessary adjuvant chemotherapy has important economic implications, as it allows healthcare resources to be allocated more efficiently to patients who are more likely to benefit from additional treatment. These findings support a more individualized approach to adjuvant therapy, where treatment decisions are based on the patient's pathological response to neoadjuvant therapy and their overall risk of recurrence.

This study has several limitations that must be acknowledged. First, the retrospective design limits control over confounding variables, and the relatively small sample size reduces statistical power. Second, imbalances in baseline characteristics, such as a higher proportion of T3 tumors and APR in the adjuvant chemotherapy group, may have influenced the results. Third, important prognostic factors, including extramural venous invasion (EMVI), tumor deposits, and circumferential margin (CRM) status, were not consistently documented, limiting the ability to fully assess recurrence and survival risks. Fourth, the lack of randomization and the variability in adjuvant chemotherapy regimens introduce selection bias and treatment heterogeneity. Finally, while follow-up was sufficient for observing trends in survival, longer-term outcomes remain unknown. These limitations highlight the need for larger, prospective, randomized studies to validate these findings.

In conclusion, our study suggests that adjuvant chemotherapy does not provide a significant survival benefit in patients with ypT0-2 N0 rectal cancer following neoadjuvant CRT and surgery, indicating that routine use may not be necessary. Avoiding overtreatment could reduce chemotherapy-related toxicity and improve resource allocation. While our study provides valuable insights into the role of adjuvant chemotherapy in downstaged rectal cancer patients, its retrospective design, monocentric, small sample size, and lack of data on certain prognostic factors underscore the need for prospective studies to confirm these results. Future research should focus on larger, multicenter trials incorporating molecular profiling and biomarkers to better tailor adjuvant therapy decisions.

## Supplementary Information

Below is the link to the electronic supplementary material.

Supplementary file1 (PDF 112 KB)

## Data Availability

No datasets were generated or analysed during the current study.
